# Radiological, Radiomics, and Metastatic Patterns Associated with Targetable Oncogenic Drivers on CT-Scan of Newly Diagnosed NSCLC Patients: A Comprehensive Radiogenomics Review

**DOI:** 10.3390/cancers18030472

**Published:** 2026-01-31

**Authors:** Letuan Phan, Sophie Cousin, Lou Andrea Sitruk, Cécile Masson--Grehaigne, Mathilde Lafon, Inès Kasraoui, Antoine Italiano, Benjamin Bonhomme, Jean Palussière, Charlotte Domblides, Nathalie Lassau, Amandine Crombé

**Affiliations:** 1 SARCOTARGET Team, Bordeaux Research Institute in Oncology (BRIC), INSERM U1312, University of Bordeaux, F-33076 Bordeaux, France; 2 Department of Medical Oncology, Bergonié Institute, F-33076 Bordeaux, France; 3 Department of Radiology, Pellegrin University Hospital, F-33076 Bordeaux, France; 4 Department of Radiology, Gustave Roussy, F-94805 Villejuif, France; 5 Département d’Innovation Thérapeutique et des Essais Précoce, Gustave Roussy, F-94805 Villejuif, France; 6 Department of Biopathology, Bergonié Institute, F-33076 Bordeaux, France; 7 Department of Radiology, Bergonié Institute, F-33076 Bordeaux, France; 8 Department of Medical Oncology, Saint-André Hospital, Bordeaux University, F-33076 Bordeaux, France; 9 Biomaps, UMR1281 INSERM, CEA, CNRS, Institut Gustave Roussy, University of Paris-Saclay, F-94800 Villejuif, France

**Keywords:** radiomic, radiogenomic, metastatic pattern, non-small cell lung cancer, lung adenocarcinoma

## Abstract

The management of non-small cell lung cancer (NSCLC), including lung adenocarcinomas (LUAD), has been revolutionized with the advent of precision oncology. While advanced cancers often carry poor prognosis, those harboring specific molecular alterations sensitive to targeted therapy (notably tyrosine kinase inhibitor [TKI]) have experienced improved response to treatment and survival outcomes. Consequently, detecting these alterations through molecular screening panel has become standard in several countries, although this necessitates high-quality tissue sampling to inform optimal therapeutic decisions. Oncologic imaging occupies a pivotal role in the routine care of patients, in particular at diagnosis, with a wealth of information gathered but underutilized, as medical imaging reflects the disease in its entirety at a given time point. Moreover, recent advancements in imaging quantitative analysis, including radiomics and artificial intelligence, could aid in better integration and understanding of this information that has been overlooked for years. Several radiological phenotypes (or radiophenotypes) have been linked to tumor genomic alterations, both in standard radiology relying on semantic features and metastatic patterns, and in radiomics. Ultimately, understanding the relationships between imaging and targetable genomic alterations via accurate imaging biomarkers could complement ambiguous tumor or liquid biopsy, detect emerging new alterations, and even substitute biopsy through ‘virtual biopsy’. During the past decade, there has been a surge in research focused on radiogenomic assessment of NSCLC and especially LUAD. However, due to the low prevalence of many oncogenic drivers, the scientific literature may lack clarity or present conflicting findings. This comprehensive review aims to provide a summary of the current state of this research, offering insights into the complex interplay between imaging and genomic alterations in lung adenocarcinoma.

## Simple Summary

Non-small cell lung cancer is not a single disease but a group of tumors potentially driven by different genetic alterations that strongly influence prognosis and treatment options. Identifying these alterations usually requires tissue biopsies, which are sometimes invasive, insufficient, or impossible to repeat over time. Medical imaging, particularly computed tomography scans, is routinely performed in all patients and may contain hidden qualitative and quantitative information reflecting underlying tumor biology. In this review, we summarize how common genetic drivers of non-small cell lung cancer are associated with specific patient characteristics, tumor appearances on imaging, and patterns of metastatic spread. We also discuss recent advances in quantitative imaging analysis, especially radiomics combined with artificial intelligence algorithms, which aims to extract biological information from medical images. Together, these tools could help improve non-invasive tumor characterization, guide molecular testing strategies, and support treatment decisions, especially when tissue samples are limited or when the disease evolves over time.

## 1. Introduction

Lung cancer remains the leading cause of cancer mortality worldwide and a major public-health burden. A recent population-based period analysis derived from the Surveillance, Epidemiology, and End Results (SEER) registry estimated a 5-year overall survival (OS) rate for non-small cell lung cancer (NSCLC) of 26.4%, falling from 68% for stage I to <6% for stage IV disease [1]—although these figures reflect outcomes from the early immunotherapy era and may therefore underestimate survival in the current treatment landscape. NSCLC comprises roughly 80–85% of lung cancers, and lung adenocarcinoma (LUAD) is the predominant histologic subtype, accounting for more than half of newly diagnosed NSCLC patients [1,2].

The discovery of somatic driver oncogenes in LUAD and the parallel development of targeted drugs have transformed the management of advanced disease. Routine adoption of high-throughput next-generation sequencing (NGS) enables simultaneous, multiplexed profiling of multiple actionable alterations, and international practice guidelines now recommend systematic molecular testing to inform targeted therapy selection, including epidermal growth factor receptor alterations [EGFR], anaplastic lymphoma kinase rearrangements [ALK], and ROS proto-oncogene 1 rearrangements [ROS1], with expanded panels to include B-Raf proto-oncogene mutations [BRAF], mesenchymal-epithelial transition factor alterations [MET], rearranged during transfection fusions [RET], neurotrophic tyrosine receptor kinase fusions [NTRK], human epidermal growth factor receptor 2 alterations [HER2], and Kirsten rat sarcoma viral oncogene homolog mutations [KRAS]) in newly diagnosed advanced non-squamous NSCLC [3,4]. Current reported mutation frequencies vary by population. EGFR alterations, for example, are observed in up to 49% of East Asian cohorts, but in only 10–16% of Western cohorts. Meanwhile, KRAS mutations predominate in Western populations (25–30% of LUAD patients), with the KRAS G12C subtype representing around 10–13% of advanced NSCLC in Western cohorts [4].

Despite these advances, tissue-based molecular testing is constrained by practical limits: biopsies may be infeasible or non-contributive, available material may not permit broad-panel NGS, and intratumoral and inter-metastatic heterogeneity create sampling bias that can produce false-negative results or fail to capture emerging resistance alterations. In practice, repeated biopsies cannot be performed in nearly 20% of patients [5], and between 5–20% of tissue samples prove inadequate for comprehensive molecular testing because of low cellularity or NGS failure [6]. Tissue NGS can fail in around a fifth of cases, even when there is sufficient material [6]. Quality-assurance studies further show that up to 40% of small biopsies may be suboptimal for full NGS, with inadequacy rates varying widely by sampling technique and site [7,8]. However, liquid biopsy (LB, i.e., the analysis of circulating tumor DNA in blood samples) offers a complementary approach and can increase the detection of actionable alterations, for example from 59.7% to 70.4% when added to tissue NGS in NSCLC. Nevertheless, a subset of patients still has an inaccessible genotype despite both assays [6]. Furthermore, real-world studies demonstrate that comprehensive profiling is not being implemented as widely as recommended by guidelines, highlighting the need for additional non-invasive approaches [3,4].

Imaging is obtained for virtually all patients at diagnosis and at each evaluation and thus represents a universal, non-invasive source of information capturing the spatial extent of disease. Standard baseline staging commonly includes contrast-enhanced (CE) thoraco-abdomino-pelvic CT-scan and brain CE-MRI (or CE-CT when MRI is unavailable), with 18F-Fluorodeoxyglucose positron emission tomography (18F-FDG PET) CT indicated in selected patients (for example, to evaluate oligometastatic disease or bone involvement to guide local therapy). These modalities are endorsed by major guidelines because they inform staging and treatment planning [9,10].

Nevertheless, radiology workflows largely rely on a limited set of qualitative semantic descriptors (i.e., explainable with human language by radiologists) and simple quantitative metrics (i.e., RECIST v1.1 and iRECIST target diameters of up to five lesions) [11,12]. This conventional approach captures only a fraction of the data present in imaging: shape analysis, internal architecture, texture and heterogeneity analyses, peritumoral environment, metastatic distribution, and patient-level metrics (cardiovascular score, body composition, bone integrity) remain under-exploited [13].

Introduced in the early 2010s, radiomics formalizes the extraction of high-dimensional, quantitative imaging features from segmented, three-dimensional volumes of interest. When combined with machine-learning algorithms, radiomics can generate predictive signatures for biological and clinical endpoints. Early foundational work established the concept and promise of radiomics. Subsequently, standardization efforts were undertaken, notably by the Image Biomarker Standardization Initiative (IBSI). These efforts were followed by the Radiomics Quality Score (RQS) and guidelines and checklists endorsed by national and international radiological societies, which addressed reproducibility and harmonization of feature definitions [13,14,15]. Furthermore, recent methodological advances include deep-learning-based ‘deep radiomics’ and multi-instance learning approaches that aim to capture intra-tumoral and inter-lesional heterogeneity across all disease sites.

In LUAD, radiogenomic research has progressed in stages. Initially, there were semantic CT descriptions associated with particular driver alterations, such as ground-glass component (GGO), solidity, spiculation, and tumor location. Next came the characterization of metastatic dissemination patterns linked to oncogenic drivers. More recently, radiomics and artificial intelligence (AI) studies have attempted to predict mutation status non-invasively. However, heterogeneity in study design, modest cohort sizes for rare drivers, variable imaging protocols, and limited external validation have produced inconsistent, fragmented literature with poor reproducibility and performance.

Because imaging is universally available at diagnosis, noninvasive imaging biomarkers have the potential to complement tissue and liquid biopsies. They can help triage cases for repeat sampling, flag probable driver alterations when tissue is insufficient, and detect spatially heterogeneous or emerging genomic events.

Accordingly, this review summarizes evidence from radiology, radiomics, and metastatic patterns to identify key actionable drivers in newly diagnosed NSCLC, with an emphasis on lung adenocarcinoma. The review also evaluates the methodological robustness and clinical applicability of these findings and identifies priorities for prospective, harmonized, multicenter radiogenomic studies.

## 2. Recommended Molecular Screening at Baseline in Patients with Newly Diagnosed Lung Adenocarcinoma

### 2.1. Indication for Molecular Screening in Latest Guidelines

In localized or locoregional NSCLC (stages I–III), baseline molecular profiling is recommended when results may influence perioperative or adjuvant management—most notably testing for EGFR mutations and ALK rearrangements, given the demonstrated benefit of adjuvant osimertinib and the emerging role of ALK inhibition in early-stage disease [16,17]. In the metastatic or unresectable setting, international guidelines uniformly endorse broad upfront molecular testing, ideally through NGS, to identify actionable genomic alterations including EGFR, ALK, ROS1, BRAF, RET, MET exon 14 skipping, KRAS G12C, NTRK, and HER2. When tissue is insufficient, unobtainable, or expected to be non-representative, LB is recommended as a complementary or alternative approach to maximize the detection of therapeutically relevant alterations [9].

### 2.2. Samples and Techniques for Molecular Screening

Tumor tissue remains the preferred and most informative material for baseline molecular profiling in NSCLC, providing adequate substrate for both immunohistochemistry (IHC) and nucleic acid-based assays. DNA-based techniques are typically employed to detect point mutations and small insertions or deletions—such as those occurring in EGFR, BRAF, and KRAS—whereas RNA-based assays are often required to reliably identify gene fusions, including ALK, ROS1, RET, and NTRK rearrangements. Comprehensive multigene testing by NGS is recommended whenever feasible, as it optimizes tissue use, minimizes the need for sequential assays, and accelerates the turnaround time of clinically actionable results.

LB serves as a complementary or alternative approach when tissue is insufficient, unobtainable, or expected to be non-representative. It offers practical advantages, including a shorter turnaround time, and may provide genomic information in patients who are unfit for or contraindicated to invasive sampling [9,18]. However, ctDNA assays may have lower sensitivity in patients with low tumor burden or intrathoracic-only disease and are therefore ideally interpreted alongside tissue-based testing.

Importantly, current National Comprehensive Cancer Network (NCCN) guidelines strongly recommend broad molecular profiling to ensure detection not only of common oncogenic drivers but also of rarer actionable alterations for which effective therapies are available or emerging. These include MET exon 14 skipping mutations or high-level MET amplification, RET rearrangements, and ERBB2/HER2 mutations, all of which have dedicated targeted agents or ongoing clinical trials supporting their clinical relevance.

### 2.3. First-Line Validated Tyrosine Kinase Inhibitor Treatments for Driver Mutations Identified on Initial Molecular Screening

In metastatic NSCLC, the identification of actionable oncogenic drivers directly determines first-line systemic therapy. For tumors harboring common *EGFR* mutations (namely exon 19 deletions and L858R substitutions), which represent approximately 10–20% of Western cases and substantially higher proportions in Asian populations, first-line osimertinib has long constituted the standard of care. Recent data support treatment intensification in selected patients. In the FLAURA2 trial, the combination of osimertinib with platinum–pemetrexed chemotherapy significantly improved outcomes compared with osimertinib alone, extending median progression-free survival (PFS) to 25.5 months vs. 16.7 months (hazard ratio [HR] 0.62; 95% confidence interval [95%CI] 0.48–0.80) and OS to 47.5 months vs. 37.6 months (HR 0.77; 95%CI 0.61–0.96) [19,20]. Similarly, the MARIPOSA trial demonstrated a significant OS benefit for amivantamab plus lazertinib over osimertinib monotherapy (HR ≈ 0.75) [21]. These results are redefining first-line standards in patients with common *EGFR* mutations, with treatment intensification generally considered for individuals with adverse prognostic features, such as baseline brain metastases or co-occurring genomic alterations.

In contrast, tumors harboring *EGFR* exon 20 insertions show intrinsic resistance to classical EGFR TKIs. The PAPILLON trial established amivantamab plus platinum–pemetrexed as a first-line option in this subgroup, demonstrating superior efficacy over chemotherapy alone [22].

Hence, the oncogenic drivers included in this review (EGFR, ALK, KRAS, ROS1, RET, MET exon 14 skipping, BRAF, HER2, and NTRK) were selected based on their clinical relevance in NSCLC, as they are currently actionable or included in guideline-recommended molecular testing panels. While these alterations differ in oncogenic dependency, biological behavior, and therapeutic sensitivity—ranging from strong driver addictions (e.g., EGFR, ALK, ROS1) to more heterogeneous or context-dependent drivers (e.g., KRAS, BRAF non-V600E)—they collectively represent the spectrum of targetable molecular alterations encountered in routine clinical practice. This review therefore focuses on comparative radiological and metastatic patterns rather than on oncogenic potency per se.

## 3. Radiological, Radiomics, and Metastatic Profiles per Main Oncogenic Drivers

Table 1 summarizes the main clinical characteristics linked to each major oncogenic driver. Table 2 provides details of their radiological features in primary and metastatic settings. It must be noted that clinical symptoms at diagnosis largely overlap across oncogenic subtypes of NSCLC and are primarily driven by tumor location, burden, and metastatic extent rather than by the underlying molecular alteration, limiting their discriminatory value for oncogenic stratification. Similarly, blood-based parameters and serum protein biomarkers were not addressed, as they currently lack specificity for oncogenic driver stratification and fall outside the radiological and radiogenomic focus of this review. To ensure consistency and avoid bias related to histology—particularly the differences between squamous cell carcinoma and adenocarcinoma—the analysis primarily focuses on LUAD, the most frequent histologic subtype in which actionable oncogenic alterations are typically identified. Additionally, we provide a summary of major radiomics studies in Table S1, defined as those including at least 100 patients and reporting an independent validation cohort, evaluating primary tumor imaging for key genomic alterations (EGFR, ALK, and KRAS).

### 3.1. EFGR-Altered NSCLCs

#### 3.1.1. General Epidemiological, Histological, and Molecular Data

*(i) Demographic characteristics. EGFR* mutations represent the most frequent actionable oncogenic driver in LUAD, with a markedly heterogeneous distribution. In Asian populations, the prevalence ranges between 40 and 50% [23]. In Western populations, the prevalence is substantially lower, averaging 8–12% in large national cohorts [24]. These mutations are strongly enriched in never-smokers and in women. Among never-smokers, *EGFR* mutations account for ~35–40% of adenocarcinomas in Europe and North America and ~50–60% in Asia, whereas frequencies among smokers are generally <10% in Western series and 15–25% in Asian cohorts. Thus, the typical phenotype is an Asian, female patient with never-smoker or light-smoker status and a preserved baseline performance status [25,26]. The age at presentation is similar to that of NSCLC patients (typically 60–70 years old). However, disease stage at diagnosis does not differ significantly between the *EGFR*-mutated and *EGFR*-wild-type groups [26].

*(ii) Histological characteristics.* EGFR-mutated tumors are almost exclusively LUADs, most commonly displaying well-differentiated patterns—particularly lepidic (44%), followed by papillary and acinar architectures [27,28]. Indeed, lepidic-predominant adenocarcinoma shows the strongest enrichment, with an EGFR mutation prevalence of 60.2% and an odds ratio (OR) of 2.53 (95%CI: 1.43–3.96) in a large imaging-pathology cohort [26]. Rare EGFR variants, such as exon 18 mutations or non-insertion exon 20 alterations, may be associated with more solid and poorly differentiated phenotypes and correspond to more aggressive clinical behavior [29].

*(iii) Molecular landscape.* Over 90% of activating EGFR mutations are exon 19 deletions (45–55%) or L858R (35–40%), with exon 18 (5–6%) and exon 20 alterations (5–10%) being less frequent; exon 20 insertions are intrinsically TKI-resistant [30]. Exon 19 deletions confer superior response and survival compared with L858R, as shown in the FLAURA trial (median PFS 18.9 vs. 10.2 months) [31,32]. Objective response rate (ORR) in treatment-naïve patients reaches 70–80%, though resistance arises, historically via T790M mutation (50–60%) or heterogeneous mechanisms under osimertinib. TP53 co-mutations (>40–50%) predict aggressive disease and earlier resistance [33]. EGFR-mutated tumors have low tumor mutational burden (TMB) (3–4 mut/Mb vs. 6 in smoking-related NSCLC), limiting response to PD-1/PD-L1 blockade [34].

#### 3.1.2. Radiological Characteristics of the Primary Lung Lesion in EGFR-Altered LUAD

**Figure 1:**
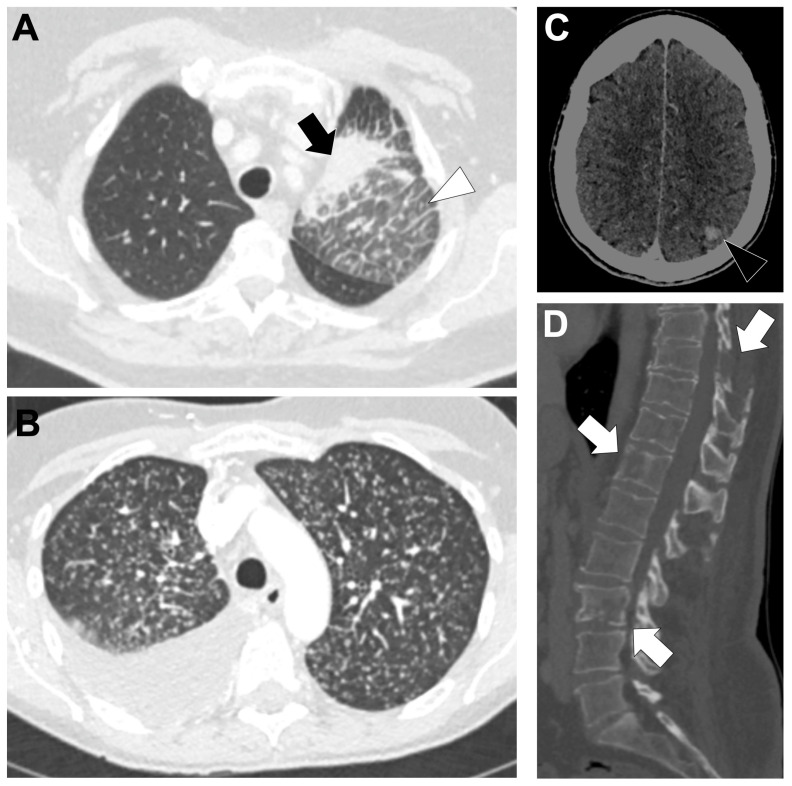
Representative baseline CT imaging findings in patients with EGFR-altered lung adenocarcinoma (LUAD). (**A**) A 71-year-old never-smoking woman presenting with a spiculated mass in the left upper lobe (black arrow), surrounded by ground-glass opacities and associated lymphangitic carcinomatosis (white arrowhead). Molecular analysis identified an exon 21 L858R EGFR mutation. (**B**–**D**) A 51-year-old woman presenting with a diffuse miliary pulmonary pattern and pleural effusion (**B**), a brain metastasis located in the left parietal cortex (black arrowhead) (**C**), and multiple lytic vertebral bone metastases (white arrows) (**D**). Molecular analysis revealed an exon 19 deletion.

Figure 1A illustrates a locally advanced EGFR-mutated LUAD. On chest CT, these tumors are typically small (mean 39.8 mm; OR = 0.81, 95%CI 0.68–0.96) with frequent GGO (18.1% vs. 9% in EGFR-wild-type, 6.5% in KRAS-mutated) and part-solid appearance, the latter being the only feature consistently enriched in EGFR-mutated tumors [25,35]. Air bronchograms (60% vs. 35%; OR = 4.56, 95%CI 2.13–9.74) and pleural retraction (65% vs. 47%; OR = 2.51, 95%CI 1.20–5.24) are frequent, while lesions are less common in smokers or emphysematous lungs (OR range 0.18–0.25) [36]. Additional independent predictors include spiculation (50.9% vs. 27.1%; OR = 2.70, 95%CI 1.54–4.75), multiple small pulmonary nodules (8.6% vs. 2.4%; OR = 7.52, 95%CI 1.44–39.17), and bubble-like lucencies (OR = 2.50, 95%CI 1.48–4.21) [25,26]. Subtype analysis shows exon 19 deletions are associated with larger, more solid tumors and more advanced disease than L858R [37,38].

#### 3.1.3. Single-Site Radiomics Signature of the Primary Lung Lesion in EGFR-Altered LUAD

Across CT-based analyses, both radiomics and DL models can distinguish EGFR-mutant from EGFR-wild-type tumors, although external-validation performance remains heterogeneous (Table S1). Jia et al. trained a random-forest radiomics model on 345 cases and validated it independently (*n* = 158), achieving AUC 0.802, increasing to 0.828 with sex and smoking added (sensitivity 60.6%, specificity 85.1%) [39]. Wang et al. developed a CNN model (training *n* = 603; external validation *n* = 241) with AUC 0.81, accuracy = 73.9%, sensitivity = 72.3%, and specificity = 75.4% [40]. A hybrid multi-task deep/radiomics model reported AUCs of 0.86 ± 0.03 and 0.80 ± 0.05 in two independent cohorts (external *n* = 131) [41]. A PET/CT radiomics meta-analysis pooling 17 studies showed validation-set AUC 0.82, with sensitivity 0.76 and specificity 0.75, while highlighting methodological heterogeneity and modest RQS scores [42]. Conversely, rigorous external testing has revealed poor reproducibility: prior CT radiomics models dropped to AUC about 0.5 on an independent NIH cohort, whereas a simple clinical model reached AUC 0.74 [43].

Overall, independent validation AUCs span 0.64–0.86, with most studies clustering around 0.78–0.83. Performance often improves when combining imaging with clinical variables, integrating peripheral and central tumor radiomics, or fusing handcrafted and deep features [44]. Yet generalizability remains inconsistent, underscoring the need for large multicenter validations, IBSI-compliant pipelines, and transparent RQS/CLAIM-aligned reporting before clinical application.

#### 3.1.4. Metastatic Pattern in EGFR-Altered LUAD

Figure 1B–D display this metastatic spreading on CT-scan. Multiple bilateral pulmonary metastases are more frequent in EGFR-mutant cases, reported in 39.2% of patients compared to wild-type (*p* = 0.0152) [45], with convergence of surrounding structures (*p* < 0.0001). Diffuse or ‘miliary’ lung metastases occur in 12–50% patients versus 3% of EGFR-wild-type cases [45,46,47,48]. EGFR-mutant tumors also show lower frequencies of nodal involvement (84% vs. 96%, *p* < 0.01), pleural lesions (40% vs. 70%, *p* < 0.01), and adrenal metastases (14% vs. 31%, *p* < 0.01) [45,46,47,48].

Brain metastases are significantly more frequent in EGFR-mutated patients: in a cohort of 1127 NSCLC patients, EGFR-mutated patients had a 31.4% incidence versus 19.7% in wild-type patients (OR = 1.86, 95%CI 1.39–2.49; *p* < 0.001) [49]. In another series of 234 LUAD patients, EGFR-mutated patients were more likely to present with brain metastases at diagnosis (*p* = 0.014) and during follow-up (*p* < 0.001), with multivariable OR = 2.51 for baseline brain metastases (*p* = 0.022) [50]. EGFR mutation was the only independent risk factor for subsequent brain metastases (HR = 3.036, *p* = 0.001), and patients with EGFR-mutated brain metastases showed longer OS than wild-type (*p* = 0.028).

Among EGFR variants, exon 19 mutations are associated with lung miliary and brain metastases, which confer adverse prognosis [48,51,52]. Exon 21 mutations are associated with more frequent liver metastases (23% vs. 7%, *p* < 0.01) [53].

Importantly, discordance in EGFR mutation status between primary tumors and metastases complicates the interpretation of metastatic patterns, with reported discordance rates ranging from 10% to 31% depending on site, cohort, and methodology [54,55].

### 3.2. ALK-Altered NSCLCs

#### 3.2.1. General Epidemiological, Histological, and Molecular Data

*(i) Demographic characteristics*. Patients with ALK-rearranged NSCLC are typically younger (median 57–59 y) than ALK-negative cases and more often never-smokers [56,57,58,59]. Indeed, younger age is independently associated with ALK positivity (OR = 0.96, 95%CI = 0.92–1.00) [36]. In a multicenter cohort (*n* = 81 ALK vs. 146 WT), 46% of ALK-positive patients were never-smokers versus 10% in ALK-negative patients [60]. Compared to EGFR-mutated patients, ALK-positive patients also seem to be younger and less likely to have a smoking history, but sex distribution remains controversial [61].

*(ii) Histological characteristics*. ALK-rearranged NSCLC almost exclusively corresponds to LUAD, showing a strong predilection for more aggressive histological subtypes. In a cohort of 2299 Chinese patients, 93 tumors (4.0%) harbored ALK rearrangements, with frequencies by main subtypes as follows: invasive adenocarcinoma variants 14.8%, solid predominant 10.3%, and micropapillary predominant 7.6% [62].

*(ii) Molecular landscape*. ALK rearrangements occur in approximately 3–7% of NSCLC patients, predominantly as EML4–ALK fusions, which drive the constitutive activation of downstream pathways including MAPK, PI3K/AKT/mTOR, and JAK/STAT, promoting tumor initiation and progression [63,64,65]. Large NGS analyses (*n* = 6576) identified ALK fusions in 343 tumors (5.2%), of which 78.4% were EML4-ALK with three main variants [66]. ALK-positive tumors are theoretically mutually exclusive with EGFR or KRAS mutations and exhibit a ‘simple’ genomic landscape characterized by low TMB, TP53 co-mutations in 20–25%, and few additional alterations, likely contributing to their sensitivity to ALK-targeted therapies [67].

#### 3.2.2. Radiological Characteristics of the Primary Lung Lesion in ALK-Altered LUAD

**Figure 2:**
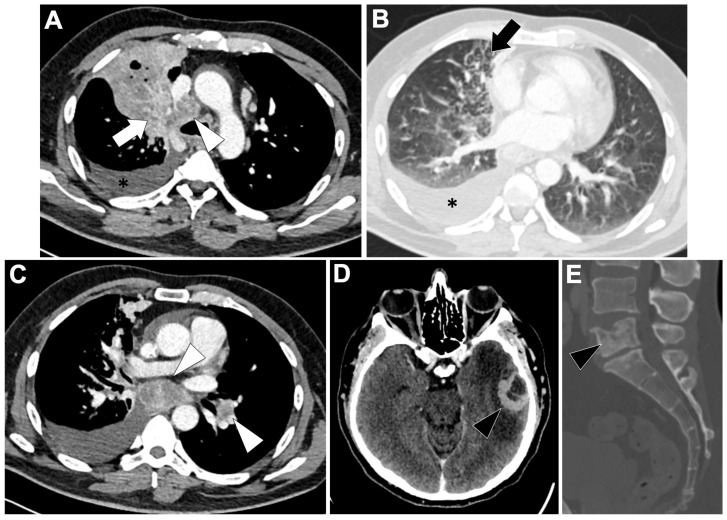
Representative baseline CT imaging findings in patients with ALK-altered lung adenocarcinoma (LUAD). (**A**) A 51-year-old never-smoking man presenting with a right middle lobe perihilar mass (white arrow) causing partial atelectasis, associated with right paratracheal lymphadenopathy (white arrowhead) and a pleural effusion (black asterisk). (**B**) Lung window images demonstrate peritumoral lymphangitic carcinomatosis (black arrow) in addition to the pleural effusion (black asterisk). (**C**) The CT scan also showed additional heterogeneous mediastinal lymphadenopathy, including subcarinal and contralateral nodes (white arrowheads). (**D**) Brain CT reveals a necrotic temporal brain metastasis (black arrowhead). (**E**) Bone window images show a mixed lytic–sclerotic metastasis involving the L5 vertebral body (black arrowhead).

Figure 2A shows a CT-scan of a locally advanced ALK-altered LUAD.

They typically present as solid, centrally located primary tumors that are often associated with bulky multifocal lymphadenopathy and lymphangitic carcinomatosis [56,57,58,59,61,68]. Pleural effusion is more frequent in ALK-rearranged patients, with reported rates of 32% versus 15% in ALK-negative tumors (OR = 2.91, 95%CI 1.25–6.80) [36], and pleural or pericardial involvement is common. Compared with EGFR-mutated tumors, ALK-rearranged lesions are more often lobulated (OR = 4.82, *p* = 0.002), exhibit lymphangitic spread (OR = 8.48, *p* = 0.002), and show higher rates of N2–N3 nodal disease (OR = 2.45, *p* = 0.049) [61]. In a meta-analysis of 3113 NSCLC patients, including 528 ALK-rearranged patients, central tumor location (OR = 2.72, *p* < 0.01), solid density (OR = 2.86, *p* < 0.01), lymphangitic carcinomatosis (OR = 3.46, *p* < 0.01), pleural effusion (OR = 1.91, *p* < 0.01), and pleural metastases (OR = 1.81, *p* < 0.01) were significantly associated with ALK positivity, whereas small tumors ≤ 3 cm were less frequent (OR = 0.57, *p* = 0.04) [58]. Predictive radiological models incorporating central location, large pleural effusion, absence of pleural tail, and age < 60 y achieved an AUROC of 0.846 in training and 0.788–0.894 in independent validation, performing best in stage ≤ IIIB tumors [56].

#### 3.2.3. Single-Site Radiomics Signature of the Primary Lung Lesion in ALK-Altered LUAD

A limited number of CT- and 18F-FDG PET/CT-based studies addressed the detection of ALK rearrangements using radiomics, and these studies are summarized in Table S1. Cohorts ranged from 124 to 526 patients, with ALK-positive prevalence ranging between 14 and 39%. Radiomic feature pre-selection or selection methods were mostly least absolute shrinkage and selection operator (LASSO) and/or correlation filter. Three studies used support vector machine classifier, three studies used logistic regression, and the last one employed random forests to discriminate between ALK mutant and wild-type patients. Overall, the median AUC was 0.829 (range: 0.680–0.890) and the median accuracy was 0.810 (range: 0.730–0.849). Furthermore, PET/CT-based models provided similar AUCs. Combining radiomics with immune markers yielded an AUC of up to 0.88 [69]. However, external validation was limited. Additionally, small cohorts and class imbalance reduced reproducibility. In summary, radiomics can moderately discriminate ALK-positive LUAD, but performance varies and is not yet operational for clinical use.

#### 3.2.4. Metastatic Pattern in ALK-Altered LUAD

Figure 2B–E show metastatic spread on a CT scan of an ALK-rearranged patient. ALK-rearranged LUADs display a distinct metastatic pattern characterized by preferential lymphatic and nodal dissemination. In a meta-analysis of 3113 NSCLC patients (528 ALK-rearranged, 17%), ALK-rearranged tumors were significantly associated with N2–N3 nodal involvement (OR = 5.63, *p* < 0.01), lymphangitic carcinomatosis (OR = 3.46, *p* < 0.01), pleural effusion (OR = 1.91, *p* < 0.01) and pleural metastases (OR = 1.81, *p* < 0.01), while bone metastases tended to be less frequent (OR = 0.44, not significant) compared to ALK-negative tumors [58]. Cohort data confirm higher incidences of brain (42% vs. 29%), pulmonary metastases (37% vs. 24%), and lymphangitic spread (7% vs. 1%) compared with ALK-wild-type patients [60]. Comparative studies against EGFR-mutated LUAD also indicate a predominance of lymphangitic carcinomatosis (OR = 8.48, *p* = 0.002) and advanced N2–N3 nodal disease (OR = 2.45, *p* = 0.049), while hematogenous dissemination such as bone metastases was less frequent [61]. This pattern suggests ALK-positive tumors spread primarily via lymphatic routes, contrasting with EGFR-mutated tumors, which show higher rates of miliary lung and bone metastases. Brain metastases also remain common [60].

### 3.3. KRAS-Altered NSCLC

#### 3.3.1. General Epidemiological, Histological, and Molecular Data

*(i) Demographic characteristics.* KRAS mutations represent the most common oncogenic drivers in NSCLC. In a large NGS cohort of 17,095 tumors, KRAS alterations were found in 27.5% patients, with a strong predominance in LUAD (32.6–37.2%) compared with squamous-cell carcinoma (4.4%) [70,71]. KRAS mutations are strongly associated with tobacco exposure: in a study of 3026 adenocarcinomas, KRAS alterations were detected in 34% of smokers versus 6% of never-smokers [72]. Median age typically ranges from the late 50 s to late 60 s, with a median age similar to wild-type NSCLC and a larger proportion of men [73].

*(ii) Histological characteristics.* KRAS-mutant NSCLC are particularly enriched in invasive mucinous adenocarcinoma, for which KRAS represents the dominant driver alteration [70,71].

*(iii) Molecular landscape.* The most frequent subtypes of KRAS mutations are G12C (40%), G12V (19%), and G12D (15%) [71]. KRAS mutations are generally mutually exclusive with other canonical driver events [70]. However, co-mutations in tumor suppressor genes are common especially with TP53 (40%) and STK11 (12–20%) [71,73]. KRAS-mutant tumors exhibit higher PD-L1 expression and elevated TMB, a relationship that persists even after adjusting for smoking status [71]. Early data suggested inferior prognosis for KRAS G12C compared with other KRAS subtypes [74], but this paradigm has shifted with the advent of targeted inhibitors. Sotorasib and adagrasib have demonstrated clinically meaningful activity in previously treated KRAS G12C-mutated NSCLC, with ORRs of 41% and 43%, respectively, and a median PFS of 6–6.5 months [75,76].

#### 3.3.2. Radiological Characteristics of the Primary Lung Lesion in KRAS-Altered LUAD

**Figure 3:**
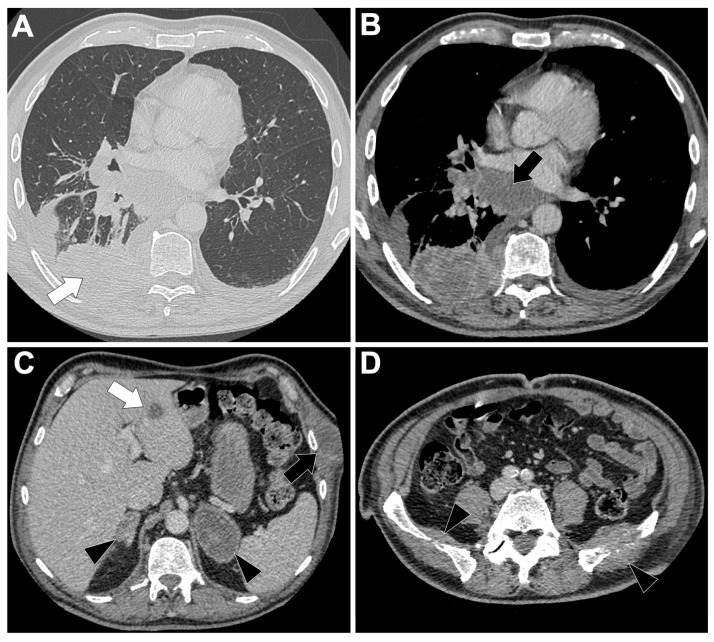
Representative baseline CT imaging findings in patients with KRAS-altered lung adenocarcinoma (LUAD). A 67-year-old man with a heavy smoking history (50 pack-years) presented with a peripheral necrotic mass in the right lower lobe (white arrow) (**A**), associated with bulky necrotic subcarinal lymphadenopathy (black arrow) (**B**). Abdominal CT images (**C**) demonstrated bilateral necrotic adrenal metastases (black arrowheads), a hepatic metastasis (white arrow), and intercostal muscle metastases (black arrow). (**D**) The patient also exhibited bilateral lytic metastases of the iliac bones (black arrowhead). Molecular analysis identified a KRAS G12C mutation.

Figure 3A,B show a CT-scan of a typical, locally advanced, KRAS-altered LUAD.

Since KRAS-positive tumors are more prevalent in smokers, the underlying lung parenchyma is more often emphysematous (25% vs. 13%, *p* = 0.03) [36]. KRAS-mutated LUADs usually present as solid lesions with minimal or absent GGO [77]. Tumors tended to show round morphology (OR = 2.40, 95%CI 1.06–5.42) and were associated with additional nodules in non-tumor lobes (OR = 1.89, 95%CI 1.03–3.50) [36]. In the multivariable predictive model developed by Rizzo et al., round shape remained more significant (27% vs. 8%, *p* = 0.009), although model performance was modest (AUC = 0.67 in the training cohort and 0.60 in the validation cohort) [36]. KRAS-mutated tumors were also larger than EGFR-mutated or wild-type lesions (mean diameter: 43.3 mm vs. 37.6 mm vs. 32.4 mm, respectively, *p* = 0.003) [25]. Spiculation was significantly linked to KRAS status (OR = 2.99, 95%CI 1.16–7.68) [78]. The KRAS G12C subtype appeared to exhibit more cavitations (13% vs. 5%, *p* = 0.04) than non-G1C mutations [79].

#### 3.3.3. Single-Site Radiomics Signature of the Primary Lung Lesion in KRAS-Altered LUAD

Only a few retrospective studies have examined the use of radiomics and deep learning to predict KRAS mutations based on initial imaging. The main studies are presented in Table S1. For instance, Wang et al. analyzed 258 patients using 18F-FDG PET/CT, with 50% KRAS prevalence in both the training (*n* = 180) and test (*n* = 78) cohorts. A radiomics-only logistic regression model achieved an AUC of 0.834, a sensitivity of 0.923, a specificity of 0.641, and an accuracy of 0.782 [80]. Dong et al. applied a multi-channel, multi-task deep learning model (CT) in 525 patients (training *n* = 363, test *n* = 162) with KRAS prevalence of 23–24%, achieving an AUC of 0.742 [81]. Shiri et al. used 18F-FDG PET/CT in 150 patients (training *n* = 82, test *n* = 68; KRAS 24%), with a stochastic gradient descent classifier, reporting AUC 0.83 [82]. Velazquez et al. combined CT radiomics with clinical features in 763 patients (training *n* = 353, test *n* = 352; KRAS 24%), achieving AUC 0.63 for radiomics alone and 0.75 when clinical data were added [83]. Overall, the median AUC for KRAS detection was 0.830 (range: 0.630–0.896), while the accuracy, reported in only two studies, ranged from 0.782 to 0.803. This corresponds to moderate-to-high accuracy, and the integration of clinical or molecular variables consistently improved model performance.

#### 3.3.4. Metastatic Pattern in KRAS-Altered LUAD

KRAS-altered LUADs display a characteristic metastatic profile dominated by intrapulmonary dissemination (Figure 3B–D). Pulmonary metastases are frequent, with multiple small lung nodules reported in 9.7% of KRAS-mutated tumors versus 2.4% in controls (adjusted OR = 7.65, 95%CI 1.18–49.50; *p* = 0.033), and overall lung metastatic involvement ranging from 35–46% across cohorts—significantly higher than in non-KRAS tumors [25,79,84]. However, KRAS-G12C tumors show fewer lung metastases than EGFR-mutated LUAD (38% vs. 67%, *p* = 0.0008) [79]. Brain metastases are common, particularly in KRAS-G12C tumors, occurring in ~42% of cases compared with 22% in fusion-driven LUAD (*p* = 0.005), and are more often solitary than in EGFR-mutated disease (median 1 vs. 4 lesions) [79,85,86]. Bone (26%) and adrenal (17%) metastases occur at intermediate frequencies without clear enrichment compared with other molecular subtypes [60,84]. In contrast, pleural involvement is relatively uncommon (15–21%), significantly lower than in fusion-positive NSCLC (21% vs. 41%, *p* = 0.01), and lymphangitic carcinomatosis is rare (≈4% vs. 39%, *p* < 0.0001) [25,79,84]. Hepatic metastases are infrequent (~11%) and negatively associated with KRAS alterations (*p* = 0.0023), while pericardial spread is exceptional [60,84]. Distant nodal metastases are slightly more common in KRAS-G12C than in EGFR-mutated LUAD (10% vs. 2%, *p* = 0.02) [79].

### 3.4. ROS1-Altered NSCLC (Figure 4)

#### 3.4.1. General Epidemiological, Histological, and Molecular Data

ROS1 rearrangements occur in approximately 1–3% of NSCLC, predominantly in LUAD (≈86–88%), and are mutually exclusive with other actionable driver mutations such as ALK, EGFR, KRAS, HER2, and BRAF [4,87,88]. ROS1-positive patients tend to be younger (median age 49–55 y), female (60–72%), of Asian origin, and never-smokers (65–72%) [89,90]. Most cases present at advanced stages (III–IV: 73–92%) [87,89]. Approved targeted therapies include crizotinib (ORR: 72%, median PFS: 19.2 months) and entrectinib (ORR: 67%, median PFS: 15.7 months); however, access to entrectinib remains heterogeneous across countries and is not routinely available in all healthcare systems, including France [4,91].

**Figure 4:**
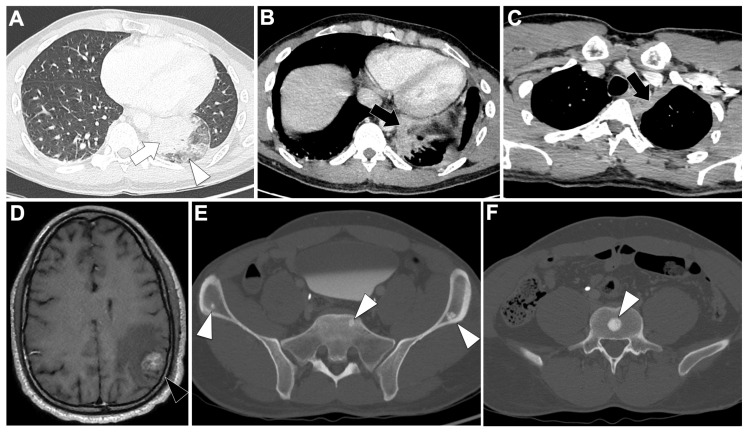
Representative baseline CT imaging findings in patients with ROS1-altered lung adenocarcinoma (LUAD). A 38-year-old man with no smoking history presented with a left lower lobe mass (white arrow) surrounded by ground-glass opacities (white arrowhead) on chest CT (**A**). On the mediastinal window, ipsilateral pleural soft-tissue infiltration was observed (black arrows) (**B**,**C**). Contrast-enhanced T1-weighted brain MRI demonstrated a heterogeneous parietal cortical metastasis (black arrowhead) (**D**). Bone-window CT images revealed multiple sclerotic bone metastases (white arrowheads) (**E**,**F**).

#### 3.4.2. Radiological Characteristics of the Primary Lung Lesion in ROS1-Altered LUAD

ROS1-positive LUAD predominantly presents as peripheral, solid nodules (88–98%) with spiculated margins in 65–71% patients [90,92]. Air bronchogram (OR = 0.05, *p* = 0.01) and pleural retraction are uncommon, while pleural effusion is relatively frequent (≈24%) [90,92]. Tumor size is generally moderate, and lymphangitic carcinomatosis is observed in 42% patients, significantly higher than in EGFR-mutant tumors (12%) [90].

#### 3.4.3. Metastatic Pattern in ROS1-Altered LUAD

ROS1-rearranged NSCLC exhibits extensive nodal involvement, including both intrathoracic and distant extrathoracic lymph nodes (47–61%), and pericardial or pleural metastases are more frequent compared with EGFR- or ALK-positive tumors [59,90]. Brain metastases are less common than in ALK or EGFR subgroups (9–19% vs. 25–40%) [88,90]. Bone metastases are predominantly sclerotic (17%), while liver and adrenal involvement is less frequent (20% and 16%, respectively) [88,89]. Multivariable logistic regression identified younger age (<56 y; OR = 0.26), lymphangitic carcinomatosis (OR = 8.77, *p* < 0.01), absence of lung metastases (OR = 0.2, *p* = 0.02), absence of brain metastases (OR = 0.06, *p* < 0.01), and distant nodal metastases (OR = 21.61, *p* < 0.01) as independent predictors of ROS1 positivity [90].

### 3.5. RET-Altered NSCLC (Figure 5)

#### 3.5.1. General Epidemiological, Histological, and Molecular Data

RET fusions represent approximately 1–2% of NSCLC [93]. RET-positive patients typically present with adenocarcinoma histology, younger age around 60 years, minimal or no smoking exposure, poorly differentiated tumors, and frequent diagnosis at advanced stages [87,94,95,96]. The most prevalent fusion partner is KIF5B (70–90%), followed by CCDC6 (10–25%) [95]. Histologically, high-grade solid non-acinar patterns have been reported as common features [97]. Selective RET inhibitors such as pralsetinib and selpercatinib show high clinical activity, achieving ORRs of 55–85% and prolonged survival compared with earlier multitargeted TKIs [98,99].

**Figure 5:**
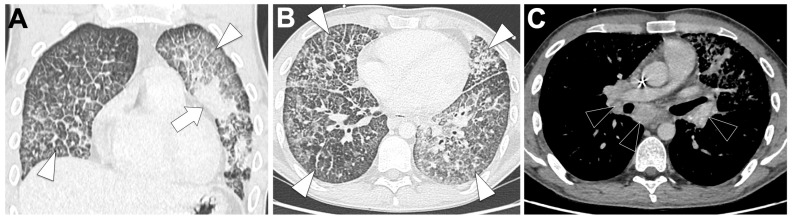
Representative baseline CT imaging findings in patients with RET-altered lung adenocarcinoma (LUAD). A 22-year-old man with a light smoking history presented with extensive bilateral lymphangitic carcinomatosis (white arrowheads) and a centrally located, spiculated solid mass in the left upper lobe (white arrow) on chest CT (**A**,**B**). On mediastinal kernel images, multiple enlarged bilateral mediastinal lymph nodes were also identified (black arrowheads) (**C**).

#### 3.5.2. Radiological Characteristics of the Primary Lung Lesion in RET-Altered LUAD

Radiologically, RET-rearranged primary tumors are typically peripheral, solid (>90%), and non-cavitated, with infrequent air bronchograms (~6%) [92,100]. Spiculated contours and mediastinal lymphadenopathy are common, while calcification and cavitation are rare [92]. Tumor size is generally around 3 cm, and imaging features substantially overlap with those of ALK- and ROS1-positive tumors, with no consistently discriminatory characteristics across fusion subtypes [100].

#### 3.5.3. Metastatic Pattern in RET-Altered LUAD

RET-fusion NSCLC frequently presents at stage IV (≈75–80%) with high extrathoracic metastatic burden [99,100]. Central nervous system (CNS) involvement is notable: approximately 20–25% of patients have brain metastases at diagnosis, and up to 45% develop CNS metastases over their disease course [95,101]. Lung metastases occur in about 50% of cases, followed by bone (≈40–45%), pleura (≈40%), lymph nodes (≈36%), and liver (≈20%) [99]. Bone metastases may be sclerotic, a pattern shared with ALK- and ROS1-rearranged cancers [100]. Lymphangitic carcinomatosis and multiple bilateral pulmonary nodules have been described in several patients, reinforcing the tendency toward diffuse thoracic spread [102].

### 3.6. MET Exon 14-Altered NSCLC (Figure 6)

#### 3.6.1. General Epidemiological, Histological, and Molecular Data

MET exon 14-altered NSCLC accounts for approximately 3% of LUAD with MET exon 14 skipping representing about 98% of MET exon 14 alterations [4]. These tumors typically arise in older patients, with median ages between 70 and 75 years [103,104], and show a slight female predominance (about 60%) [103]. Smoking history is variable across cohorts, ranging from predominantly never-smokers (≈70%) [105] to mixed populations with >50% smokers [103,104]. Most patients present with advanced disease, with stage IV reported in 40–76% patients [103]. Targeted therapies include crizotinib and the selective MET TKIs capmatinib and tepotinib (ORR 46%) [4,106,107].

**Figure 6:**
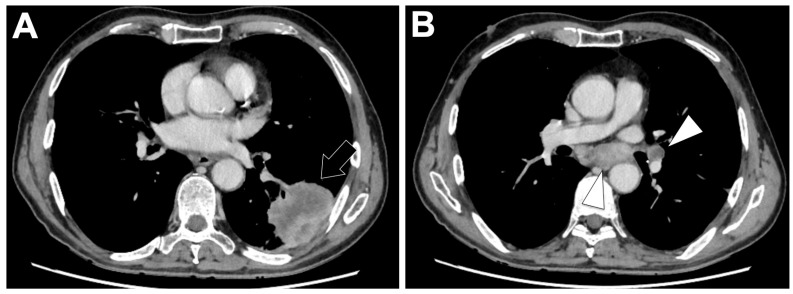
Representative baseline CT imaging findings in patients with MET exon 14 skipping lung adenocarcinoma (LUAD). A 76-year-old man with a history of tobacco use presented with a large, necrotic, peripheral, lobulated mass in the left lower lobe (black arrow) (**A**) accompanied by multiple necrotic mediastinal lymphadenopathies (white arrowheads) (**B**).

#### 3.6.2. Radiological Characteristics of the Primary Lung Lesion in MET Exon 14-Altered LUAD

Radiologic studies, relying all on retrospective series, report large primary tumors, with solid masses >3 cm in 63% of cases [103]. Lesions are predominantly peripheral (≈74%) and often located in the upper lobes (≈70%) [103]. Pure GGO is uncommon (≈7%), whereas mixed GGO–solid morphology is observed in about 25% tumors [103]. Lobulated margins are frequent (63%) while cavitation, air bronchogram, or cystic changes are rare (3–5%) [103]. Another series reported large (median 45 mm), bulky, necrotic tumors with peripheral enhancement and frequent invasion of adjacent structures [104].

#### 3.6.3. Metastatic Pattern in MET Exon 14-Altered LUAD

Among patients with available staging data, 40–76% present with stage IV disease, and extrathoracic metastases occur in up to 82% of metastatic patients [103]. Bone metastases (≈41%), typically lytic, are common, followed by brain (≈20%) and adrenal metastases (≈20%) [103]. Large metastatic lesions frequently exhibit necrotic components, reported in 73% of advanced-stage cases [104]. Overall, MET exon 14-mutated tumors display a metastatic pattern marked by high rates of extrathoracic involvement and a tendency toward necrotic metastatic deposits.

### 3.7. BRAF-Altered NSCLC (Figure 7)

#### 3.7.1. General Epidemiological, Histological, and Molecular Data

BRAF mutations are detected in approximately 2–4% LUADs, with the V600E substitution representing a significant subset of BRAF alterations. This substitution has been reported as 1–2% of unselected NSCLC overall (i.e., approximately 50% of BRAF-mutant patients). BRAF variants are functionally classified into three groups: class I (including V600), class II, and class III. This taxonomy has prognostic and therapeutic implications [108,109,110]. Clinically, BRAF-mutant patients are typically in their mid-sixties, are evenly distributed by sex, and have a high prevalence of smoking history. However, V600E cases include a higher proportion of light smokers [111,112]. The BRAF-targeted combination of dabrafenib and trametinib has achieved high response rates (63–64% ORR and 9.7–10.9 median PFS months) and is now the standard regimen for V600E-mutant NSCLC [4,113]; however, in current clinical practice, it is most often administered in the second-line setting, whereas first-line management relies on chemo-immunotherapy.

**Figure 7:**
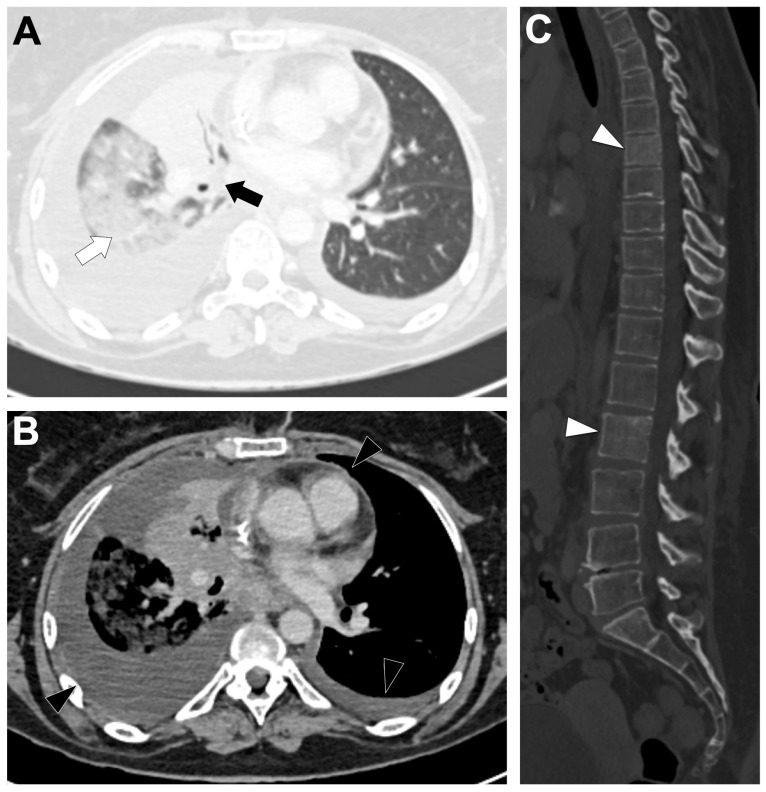
Representative baseline CT imaging findings in patients with BRAF exon 15 V600E (class 1) substitution lung adenocarcinoma (LUAD). A 43-year-old woman with a heavy smoking history presented with a central right upper lobe tumor (black arrow) causing segmental atelectasis and surrounded by ground-glass opacity (white arrow) (**A**). Bilateral pleural and pericardial effusions with metastatic spread were observed (black arrowheads) (**B**). On bone-window imaging, sclerotic vertebral metastases were identified (white arrowheads) (**C**).

#### 3.7.2. Radiological Characteristics of the Primary Lung Lesion in BRAF-Altered LUAD

BRAF-mutant primary tumors are most often solid, peripheral, and frequently spiculated, with a median lesion size near 34 mm; roughly half present as discrete masses and 77% are solid on CT in the rare retrospective cohorts [57,111]. Importantly, no CT feature or combination of features seem to reliably discriminate BRAF-mutant from BRAF-wild-type tumors across studies as radiologic appearance overlaps substantially with other molecular subtypes [57,111].

#### 3.7.3. Metastatic Pattern in BRAF-Altered LUAD

BRAF-mutant NSCLC commonly presents at advanced stage (reported stage IV proportions ranging from 43% to 64%), with BRAFV600E showing a higher frequency of stage IV presentation in some cohorts (56% vs. 43% for all BRAF) [110,114]. Functional class influences dissemination: class I (V600) tumors are more likely to have intrathoracic spread and pleural involvement, whereas classes II–III (non-V600) show a higher propensity for intra-abdominal metastases [115]. Brain metastases are less frequent with V600E than with some non-V600 classes (≈9% for V600E vs. 26% class II vs. 44% class III in one series), highlighting heterogeneity in metastatic tropism by functional class [110].

### 3.8. HER2-Altered NSCLC (Figure 8)

#### 3.8.1. General Epidemiological, Histological, and Molecular Data

HER2 (ERBB2) alterations typically consist of in-frame exon 20 duplication/insertions that induce constitutive activation of downstream PI3K/AKT/mTOR and MEK/ERK pathways and promote uncontrolled cellular proliferation [116,117]. Clinically, HER2-mutant NSCLC tends to occur in slightly younger patients with a median age around 60 years and shows a strong female predominance (about 72%) together with a high prevalence of never-smokers [118]. The histologic subtype is overwhelmingly LUAD.

**Figure 8:**
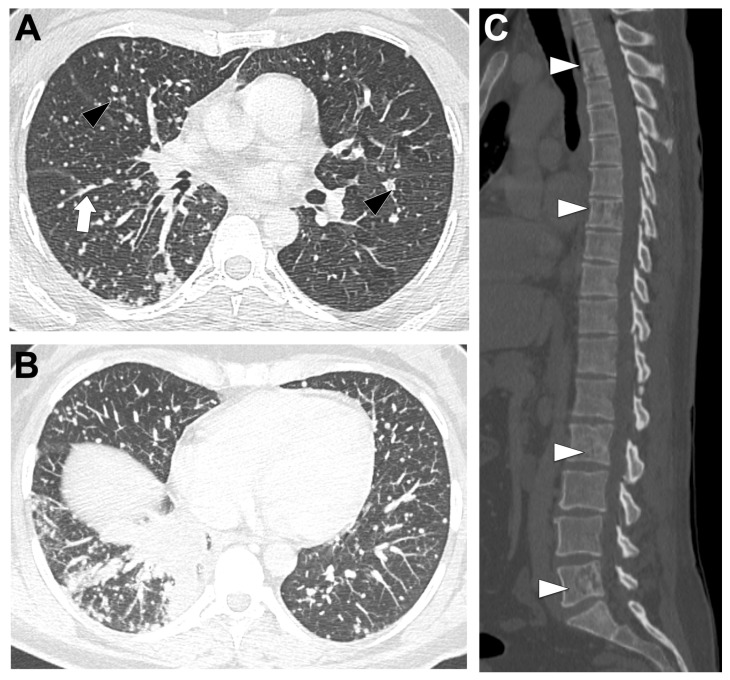
Representative baseline CT imaging findings in patients with HER2-altered lung adenocarcinoma (LUAD). A 50-year-old never-smoking woman presented with multiple pulmonary nodules (black arrowhead) and a pleural nodule along the fissures (white arrow) (**A**,**B**). Bone-window CT revealed multiple sclerotic and lytic vertebral metastases (white arrowheads) (**C**). Molecular analysis identified an in-frame exon 20 HER2 substitution.

#### 3.8.2. Radiological Characteristics of the Primary Lung Lesion in HER2-Altered LUAD

Imaging studies describe HER2-mutant LUAD as predominantly peripheral, solid nodules measuring approximately 2–3 cm, often with spiculated margins and a high incidence of pleural abnormalities. Pleural tags (≈74%) and pleural retraction (≈58%) are frequently reported, reflecting a propensity for localized invasive behavior [118]. Lymph-node involvement is common, consistent with early regional spread. Additional observations include the possibility of GGO and a larger proportion of small T1a tumors (<1 cm: 14.8% vs. 1.7% in HER2-wild-type) in some cohorts.

#### 3.8.3. Metastatic Pattern in HER2-Altered LUAD

HER2-mutated NSCLC demonstrates a significant prevalence of stage IV disease, reported in about half of patients [119]. Metastatic dissemination often includes multiple intrapulmonary nodules and both satellite lesions (24%) and distant lung nodules (28%), including miliary pattern, as well as a notable rate of excavated lesions. Extrapulmonary metastases are commonly observed in the brain and bone, with many patients presenting with multiorgan involvement at diagnosis [117].

### 3.9. NTRK-Altered NSCLC (Figure 9)

#### 3.9.1. General Epidemiological, Histological, and Molecular Data

NTRK1/2/3 gene rearrangements remain exceptionally rare in NSCLC, consistently reported in <1% patients (0.23% in a large U.S. registry analysis) [120,121]. Most tumors are LUADs (≈80%), and patients tend to be younger than typical NSCLC populations (median age ≈ 48 years), with a substantial proportion of never-smokers [121]. These tumors exhibit marked oncogene dependence, as illustrated by the high activity of selective TRK inhibitors (larotrectinib, entrectinib), with a reported ORR of 75% [121].

**Figure 9:**
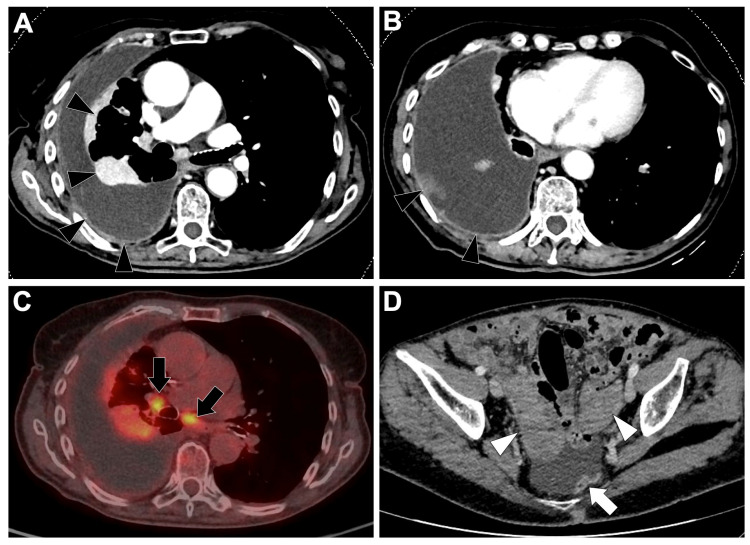
Representative baseline CT imaging findings in patients with NTRK-rearranged lung adenocarcinoma (LUAD). A 76-year-old never-smoking woman presented with a large right pleural effusion and pleural metastatic deposits (black arrowheads) (**A**,**B**). 18F-FDG PET/CT demonstrated metabolically active mediastinal lymphadenopathies (black arrows) (**C**). Pelvic CT revealed bilateral ovarian metastases (white arrowheads), peritoneal effusion, and peritoneal tumor deposits (white arrow) (**D**).

#### 3.9.2. Radiological Characteristics of the Primary Lung Lesion in NTRK-Altered LUAD

Available imaging data remain extremely limited due to the rarity of NTRK fusions, and no reproducible primary-tumor radiological signature has been established. In the largest dedicated cohort to date, primary lesions were heterogeneous in size and location without a consistent discriminative pattern compared with other oncogenic drivers [121]. Current radiology-based assessments therefore consider NTRK-rearranged NSCLCs radiologically nonspecific, reinforcing the need for molecular testing rather than imaging-based suspicion [122,123].

#### 3.9.3. Metastatic Pattern in NTRK-Altered LUAD

Limited retrospective data suggest a tendency toward advanced-stage presentation. In the largest series, 73% of patients had stage IV disease at diagnosis [121]. Metastatic spread appears to primarily involve lymph nodes (72.7%), followed by bone (54.5%), the pleura (45.5%), additional pulmonary sites (45.5%), and the brain (36.4%) [121]. Although the number of cases is insufficient to define a definitive metastatic signature, this pattern resembles that of other fusion-driven adenocarcinomas in that it suggests multisite dissemination.

### 3.10. Comparative Analysis of Oncogenic Addictions in NSCLC

#### 3.10.1. According to Patient-Related Factors (Table 1)

Differences in demographic and clinical profiles are observed across oncogenic drivers. EGFR, HER2, MET exon 14 skipping, and ROS1 alterations are more prevalent in women. ALK, ROS1, RET, HER2, and NTRK rearrangements typically occur in younger patients compared with wild-type NSCLC, whereas MET exon 14 skipping defines the oldest molecular subgroup, most often diagnosed in patients older than 70 years. EGFR and ROS1 alterations are enriched in patients of Asian ancestry. EGFR, ALK, ROS1, RET, HER2, and NTRK alterations are strongly associated with never- or light-smoking status, in contrast to KRAS- and BRAF-mutated NSCLCs, which predominantly arise in current or former smokers.

#### 3.10.2. According to Local Radiological Characteristics of the Primary Tumor (Table 2)

At the lower stage, EGFR-mutant tumors more frequently present as smaller lesions with ground-glass components, air bronchograms, and lepidic growth patterns. In contrast, KRAS-mutant tumors are typically solid, larger, rounder, and more often associated with emphysema and spiculation. ALK, ROS1, and RET rearranged tumors show largely overlapping phenotypes: predominantly solid, peripheral masses with frequent lymphadenopathy and limited ground-glass opacity, although ROS1- and RET-positive tumors show higher rates of lymphangitic carcinomatosis and nodal involvement. MET exon 14-altered tumors often appear as large, solid, peripheral masses with necrotic components and lobulated contours. HER2-mutant tumors tend to be smaller, peripheral, spiculated lesions with pleural tags and signs of local invasion, occasionally retaining ground-glass features. BRAF-mutant NSCLCs lack a distinctive radiological signature, although class I (V600E) tumors are most often solid and peripheral. NTRK-rearranged tumors currently have no established discriminative imaging phenotype due to extreme rarity.

#### 3.10.3. According to Metastatic Dissemination Patterns (Table 2)

CNS involvement is a hallmark of fusion-driven NSCLC, being particularly prominent in ALK-rearranged tumors, frequent in RET-rearranged disease, and less common at baseline in ROS1-rearranged NSCLC, while KRAS- and MET exon 14-altered tumors show intermediate rates; EGFR-mutant tumors often present with multiple brain lesions. Pulmonary dissemination, including intrapulmonary metastases and satellite nodules, is especially enriched in EGFR- and KRAS-mutant NSCLC and represents a common route of spread in BRAF V600E disease. Pleural involvement, frequently associated with effusions and lymphangitic spread, is characteristic of EGFR- and ALK-driven tumors and is also observed in ROS1- and MET exon 14-altered NSCLC, while being less frequent in KRAS-mutant disease. Lymph node metastases, including distant nodal stations, are particularly prominent in fusion-driven tumors (ALK, ROS1, RET, NTRK), often co-occurring with pulmonary and pleural dissemination. Skeletal metastases are common in ALK-, RET-, MET exon 14-, and NTRK-altered NSCLC, and are frequently sclerotic in fusion-driven subtypes. Abdominal dissemination, including liver, adrenal, and peritoneal involvement, is relatively uncommon in EGFR- and fusion-driven tumors but is more frequently observed in KRAS-mutant and non-V600E BRAF-mutant NSCLC. Overall, although certain organ tropisms and combinations recur across molecular subtypes, metastatic patterns remain largely overlapping and insufficiently specific to reliably infer the underlying oncogenic driver on radiological grounds alone.

## 4. Conclusions

This review summarizes the demographic, clinical, radiological, and metastatic characteristics of NSCLC according to major oncogenic drivers, including EGFR, ALK, KRAS, ROS1, RET, MET exon 14, BRAF, HER2, and NTRK. Driver alterations are often enriched in non-smokers, younger patients, or specific ethnic groups, and show organ-specific metastatic tropisms; yet, this review highlights that substantial heterogeneity and overlap persist. Putative biological mechanisms may underlie some of the observed imaging and metastatic patterns, including differences in tumor mutational burden, growth dynamics, and organ-specific metastatic tropism. Oncogene-driven tumors in never-smokers tend to exhibit lower genomic complexity, which may translate into more homogeneous imaging features and preferential intrathoracic spread, whereas smoking-associated alterations are often linked to greater heterogeneity and broader dissemination. These relationships remain largely correlative and require formal validation in dedicated mechanistic studies.

Therapeutic advances, including highly selective TKIs, combination strategies with immunotherapy, and targeting of uncommon alterations such as MET exon 14 skipping or NTRK fusions, have significantly improved outcomes. Yet, precise patient selection remains critical, especially in the context of co-mutations, acquired resistance, or limited tissue and non-contributive or too-expensive liquid biopsies.

Radiomics offer promising complementary approaches to tissue-based diagnostics. ‘Virtual biopsies’ could identify driver alterations with relatively high confidence, particularly when tissue is scarce or biopsy results are inconclusive, and may allow longitudinal monitoring of emerging mutations. Multi-site radiomics can quantify intra-patient inter-tumor heterogeneity, which tends to be lower in non-smoker tumors harboring oncogenic drivers due to their lower tumor mutational burden [124,125,126]. Integration with deep learning methods, such as convolutional neural networks and multiple-instance learning, may further enhance predictive performance. Moreover, combining imaging with liquid biopsy could ultimately enable a comprehensive, minimally invasive tumor fingerprint, supporting early detection, therapeutic stratification, and dynamic resistance monitoring.

However, several challenges remain, including variability introduced by co-mutations, temporal evolution of molecular profiles, heterogeneous study populations, radiomics methodologies, and comparisons with wild-type cancers or between subgroups of oncogenic drivers, which complicate the definition of robust radiological and radiomic patterns. Standardized reference cohorts and multi-institutional validation will be essential for clinical translation.

In conclusion, while NSCLC driver mutations exhibit recurrent demographic, clinical, and radiological patterns, integrating clinical, radiological, radiomics, and liquid biopsy holds substantial potential to refine non-invasive tumor profiling, guide precision therapy, and monitor tumor evolution dynamically.

## Figures and Tables

**Table 1:** Typical demographic features associated with the main oncogenic drivers in non-small cell lung cancer. Abbreviation: WT: wild type.

Characteristics	EGFR	ALK	KRAS	ROS1	RET	MET	BRAF	HER2	NTRK
**Alteration frequency**	From 8–12% in Western population to 40–50% in Asian population	4–5%	27.5%	1–3%	1–2%	3%	2–4%	1–4%	<1%
**Most frequent alteration**	Exon 19 deletion or L858 alteration	ALK fusion (EML4-ALK)	G12C mutation	ROS1 fusion (CD74-ROS1)	RET fusion (KIF5B-RET)	MET Exon 14 skipping	(Class I) V600E substitution	Mutation: Exon 20 in frame substitution	NTRK1/2/3 gene fusion
**Age**	60–70 years (similar to WT)	Younger patients	60–70 years (similar to WT)	Younger patients (45–55 years)	Younger patients (<60 years)	Older patients (>70 years)	60–70 years (similar to WT)	Slightly younger (about 60 years)	Younger patients (48 years)
**Sex**	Women predominance	-	-	Women predominance	-	Women predominance	-	Women predominance	-
**Tobacco addiction**	Never/light smokers	Never/light smokers	Smokers	Never/light smokers	Never/light smokers	-	Smokers	Never smokers	Never smokers
**Ethnic background**	Asian origin	-	-	Asian origin	-	-	-	-	-

**Table 2:** Typical local radiological features and metastatic patterns associated with the main oncogenic drivers in non-small cell lung cancer.

Characteristics	EGFR	ALK	KRAS	ROS1	RET	MET	BRAF	HER2	NTRK
**Local characteristics**									
**Typical**	Rather small lesions, GGO, air bronchogram, spiculated margins and pleural retraction	Solid, central, lobulated tumors with pleural/pericardial effusion	Solid tumors with round morphology and spiculated margins	Peripheral solid tumors with spiculated margins	Peripheral solid tumors with spiculated margins	Large, lobulated, peripheral tumors—frequently necrotic	Peripheral solid tumors with spiculated margins	Peripheral, solid, nodules with spiculated margins and frequent pleural tags and retraction reflecting infiltrating/invasive pattern	Non discriminative feature
**Atypical**	-	-	GGO	Air bronchogram, pleural retraction	Cavitation, air bronchogram	GGO, cavitation, air bronchogram	-	-	-
**Metastatic patterns**									
**Typical**	Multiple bilateral pulmonary metastases/miliary, brain and bone metastases	Pleural metastases, lymphangitic carcinomatosis, bulky lymphadenopathy	Lung, brain, bone and adrenal metastases	Lymphangitic carcinomatosis, distant intra/extrathoracic nodal metastases, and sclerotic bone metastases	Brain, bone (possibly sclerotic), pleural, nodal and liver metastases	Bone metastases (lytic), brain and adrenal (necrotic) metastases	Heterogeneous patterns depending on alteration classes	Lymph node, brain, bone and lung metastases (multiple nodules, miliary)	Lymph nodes, bone, pleural, pulmonary and brain metastases
**Atypical**	Nodal involvement, pleural lesions, adrenal metastases	Miliary	Pericardial, pleural and liver metastases, lymphangitic carcinomatosis	Brain, pleural, pericardial and lung metastases	-	-	-	-	-
**By anatomical location**									
**Brain**	+	+	+	-	+	+	Class II–III: +	+	+
**Lung metastases**	+	-	+	-			Class I: +	+	+
**Pleura**	-	+	-	-	+		Class I: +		+
**Pericardium**		+	-	-			Class I: +		
**Lymph nodes**	-	+		+	+			+	+
**Lymphangitic carcinomatosis**		+	-	+					-
**Peritoneal carcinomatosis**									-
**Liver**			-		+	+	Class II–III: +		
**Adrenal**	-		+			+	Class II–III: +		
**Bone**	+		+	+	+	+		+	+

NOTE—Abbreviations: GGO: ground glass opacity, +: enriched, -: depleted.

## Data Availability

Not applicable.

## Supplementary Materials

The following supporting information can be downloaded at: https://www.mdpi.com/article/10.3390/cancers18030472/s1, Table S1: Summary of the main single-site radiomics studies investigating the development of radiomics model to predict oncogenic alterations in lung adenocarcinoma.

## Abbreviations

The following abbreviations are used in this manuscript:

18F-FDG PET/CT	18-fluorodeoxyglucose positron emission tomography/computed tomography
AI	artificial intelligence
ALK	anaplastic lymphoma kinase rearrangements
BRAF	B-Raf proto-oncogene mutations
CE	contrast-enhanced
95%CI	95% confidence interval
ctDNA	circulating tumor DNA
EGFR	epidermal growth factor receptor alterations
GGO	ground-glass opacity
HER2	human epidermal growth factor receptor 2 alterations
HR	hazard ratio
IBSI	Imaging Biomarker Standardization Initiative
IHC	immunohistochemistry
KRAS	Kirsten rat sarcoma viral oncogene homolog mutations
LUAD	lung adenocarcinoma
MET	mesenchymal-epithelial transition factor alterations
NGS	next-generation sequencing
NTRK	neurotrophic tyrosine receptor kinase fusions
NSCLC	non-small cell lung cancer
ORR	objective response rate
PFS	progression-free survival
RET	rearranged during transfection fusions
RQS	radiomics quality score
ROS1	ROS proto-oncogene 1 rearrangements
TMB	tumor mutational burden
TKI	tyrosine kinase inhibitor
OS	overall survival
