# Supplementary material for: Radiological, Radiomics, and Metastatic Patterns Associated with Targetable Oncogenic Drivers on CT-Scan of Newly Diagnosed NSCLC Patients: A Comprehensive Radiogenomics Review
Source: Cancers (Basel). 2026 Jan 31;18(3):472. doi: 10.3390/cancers18030472 (PMC12896922; doi:10.3390/cancers18030472)
Supplement: Supplementary file 1 [file cancers-18-00472-s001.zip › cancers-4102789-supplementary.pdf]

cancers

MDPI

Supplementary Material

Table S1. Summary of the main single-site radiomics studies investigating the development of radiomics model to predict oncogenic alterations in lung adenocarcinoma.

Predicted Alteration	Stage	Article Reference	Modality	N Patient (Training/Test)	Alteration Proportion (Training/Test)	RF Number (Radiomics Software)	Selection Methods	Best ML Algorithm	Performances of Radiomics Only Models	Performances of Combined Radiomics Models
ALK	I–IV	Chang C et al., Front. Oncol., 2021 (PMID: 33738250)	18F-FDG PET/CT	526 (367/159)	15%/33%	402 (AK software)	LASSO/MRMR	Linear combination	AUC = 0.86; ACC = 0.809; Se = 0.800; Sp = 0.844	AUC = 0.88; ACC = 0.86; Se = 0.625; Sp = 0.94
ALK	I–IV	Chen W et al., Cancer Imaging, 2025 (PMID: 40083024)	CT scan	505 (156/349)	22%/21%	2084 (Pyradiomics)	VAR/SelectKBest/LASSO/backward stepwise Logistic Regression	SVM	AUC = 0.811; ACC = 0.772	AUC = 0.849
ALK	I–IV	Hao P et al., Front. Oncol., 2022 (PMID: 36338735)	CT scan	193 (154/39)	35%	NA (Pyradiomics)	LASSO	SVM	AUC = 0.890; ACC = 0.849; Preci = 0.932; F1 = 0.747; Recall = 0.630	AUC = 0.914; ACC = 0.849; Preci = 0.932; F1 = 0.747; Recall = 0.630
ALK	I–IV	Ma D,N et al., Onco. Targets Ther., 2020 (PMID: 32764984)	CT scan	124 (87/37)	34%	NA (Pyradiomics)	Corr filter/VAR/RFE	SVM	AUC = 0.829; Se = 0.769; Sp = 0.833; PPV = 0.714; NPV = 0.870; ACC = 0.811	-
ALK	I–IV	Song L et al., Front. Oncol., 2020 (PMID: 32266148)	CT scan	335 (268/67)	34%/33%	1218 (Pyradiomics)	Univar test/DBSCAN/RFE	Logistic Regression	AUC = 0.800; ACC = 0.730; Se = 0.730; Sp = 0.73	AUC = 0.880; ACC = 0.790; Se = 0.820; Sp = 0.780
EGFR	I–III	Chang C et al., Eur. Radiol., 2021 (PMID: 33544167)	18F-FDG PET/CT	583 (409/174)	51%/51%	402 (AK software)	LASSO/MRMR	Linear combination	AUC = 0.75; ACC = 0.720; Se = 0.660; Sp = 0.770	AUC = 0.810; ACC = 0.800; Se = 0.880; Sp = 0.750
EGFR	I–IV	Chen Q et al., Onco	CT scan	233 (176/57)	55%/56%	2300 (Radi-	Random Forest-BFE	Logistic Regression	AUC = 0.765	AUC = 0.759

		Targets Ther., 2022 (PMID: 35669165)				omics in Rayplus)	selection		
EGFR	I–III	Dong Y et al., Quant. Imaging Med. Surg., 2022 (PMID: 35502390)	CT scan	132 (87/45)	57%/56%	1287 (Pyradiomics)	Univar test/Corr filter/Random forest (Boruta)	Logistic Regression	AUC = 0.800	AUC = 0.798; Se = 0.842; Sp = 0.654
EGFR	-	Feng Y et al., Front. Pharmaco.l, 2022 (PMID: -)	CT scan	168 (151/17)	74%	1409 (Pyradiomics)	VAR/LASSO	Ensemble model (Rand forest/XGBOOST/Log reg)	AUC = 0.865; ACC = 0.823	-
EGFR	I–IV	Gao J et al., EJNMMI Res., 2023 (PMID: 37014500)	18F-FDG PET/CT	515 (404/111)	60%/56%	1781 PET; 1781 CT (Pyradiomics)	VAR/Univar test/LASSO	Random forest	AUC = 0.726; ACC = 0.685; Se = 0.643; Sp = 0.756	AUC = 0.730; ACC = 0.712; Se = 0.786; Sp = 0.585
EGFR	I–IV	He R et al., Cancers, 2022 (PMID: 36230590)	CT scan	758 (530/228)	48%	NA (Pyradiomics)	LASSO/PCA/Shapley	Random Forest	AUC = 0.650; ACC = 0.610	AUC = 0.910; ACC = 0.832
EGFR	IIIB–IV	Hong D et al., Front. Oncol., 2020 (PMID: 32082997)	CT scan	201 (140/61)	50%/51%	396 (AK software)	LASSO	Logistic Regression	AUC = 0.851	-
EGFR	-	Huang W et al., Front. Pharmacol., 2022 (PMID: 35571081)	18F-FDG PET/CT	194 (138/57) = 195	46%/56%	4306 RFs and 2048 CNN	Univar test/LASSO	Scoring + Logistic Regression	AUC = 0.790; ACC = 0.720; Se = 0.530; Sp = 0.960	AUC = 0.850; ACC = 0.820; Se = 0.780; Sp = 0.880
EGFR	-	Huang X et al., Front. Oncol., 2022 (PMID: 35186727)	CT scan	1074 (770/304)	51%	NA (Pyradiomics + CNN)	Mutual information filter	LGBM	AUC = 680	AUC = 0.751
EGFR	I–IV	Huo J,W et al., Front. Oncol., 2022 (PMID: 36059655)	CT scan	608 (487/121)	51%/50%	919 (Pyradiomics)	LASSO	Logistic Regression	-	AUC = 0.886; ACC = 0.810; Se = 0.902; Sp = 0.717
EGFR	I–IV	Jia T,Y et al., Eur. Radiol., 2019 (PMID: 30778717)	CT scan	503 (345/158)	61%	440 (NA)	Univar test/Random forest	Random forest	AUC = 0.802; Se = 0.606; Sp = 0.851	AUC = 0.828; ACC = 0.772; Se = 0.747; Sp = 0.791
EGFR	I–IV	Jiang M et al., Front. Oncol., 2022 (PMID: 36052262)	CT scan	692 (514/178)	51%/51%	2153 (Pyradiomics)	Univar test/VAR/MRMR	SVM	-	AUC = 0.741; ACC = 0.708; Se = 0.622; Sp = 0.796; F1 score = 0.683

EGFR	I–IV	Kawazoe Y et al., J. Appl. Clin. Med. Phys., 2023 (PMID: 37002910)	CT scan	164 (120/44)	50%/48%	1046 (Pyradiomics)	SelectKbest/LASSO	LGBM	AUC = 0.796	AUC = 0.734
EGFR		Kawazoe Y et al., Phys. Eng. Sci. Med., 2023 (PMID: 36787023)	CT scan	172 (120/52)	/	1046 (Pyradiomics)	SelectKbest/LASSO	Logistic Regression	AUC = 0.667	AUC = 0.732
EGFR	I–IV	Kim S et al., Scientific Reports, 2024 (PMID: 38195717)	CT scan	1280 (847/433)	38%/30%	512 (CNN)	Deep learning	EfficientNet b7	AUC = 0.780; ACC = 0.707	AUC = 0.704; ACC = 0.644
EGFR	I–IV	Li S et al., Med. Phy., 2020 (PMID: 32416013)	CT scan	438 (326/112)	58%/43%	496 (IBEX)	LASSO	Logistic Regression	AUC = 0.760	AUC = 0.790; ACC = 0.720
EGFR	I–II–III	Li S et al., Korean J. Radiol., 2022 (PMID: 36047542)	18F-FDG PET/CT	179 (125/54)	58%/59%	1316 PET; 1316 CT (Artificial Intelligence Kit)	VAR/Corr filter/Univar test/Stepwise	Logistic Regression	AUC = 0.804; ACC = 0.741; Se = 0.719; Sp = 0.773	AUC = 0.837; ACC = 0.741; Se = 0.781; Sp = 0.682
EGFR	I–IV	Li X,Y et al., J. Thorac. Dis., 2018 (PMID: 30746208)	CT scan	1010 (810/200)	50%	440 (RFs + CNN)	Univar test	Random forest + CNN features linear combination	AUC = 0.811; Se = 0.804; Sp = 0.763	AUC = 0.834; Se = 0.822; Sp = 0.742
EGFR	-	Liu G et al., Transl. Lung Cancer Res., 2020 (PMID: 32953499)	CT scan	263 (210/53)	68%	1672 (Syngo-Via VB10b platform)	Corr filter/MRMR	Logistic Regression	AUC = 0.700; ACC = 0.642; Se = 0.828; Sp = 0.417	AUC = 0.760; ACC = 0.755; Se = 0.903; Sp = 0.546
EGFR	II–IV	Liu Q et al., Transl. Lung Cancer Res., 2020 (PMID: 32676319)	18F-FDG PET/CT	148 (111/37)	51%	1470 CT; 100 PET (Pyradiomics)	Univar test/VIF/Random forest/Univar Logistic Regression	XGBoost Linear combination	AUC = 0.870	-
EGFR	I–IV	Liu Y et al., Clin. Lung Cancer, 2016 (PMID: 27017476)	CT scan	298 (Bootstrap)	46%	219 (NA)	Corr filter/Univar test/Backward selection	Logistic Regression	AUC = 0.647	AUC = 0.709
EGFR	I–IV	Lu X et al., Phys. Med. Biol., 2020 (PMID: 31978901)	CT scan	104 (83/21)	61%/62%	Pyradiomics	LASSO	Logistic Regression	AUC = 0.837	AUC = 0.894
EGFR	III–IV	Lu J et al., Dis. Markers, 2022 (PMID: 35578691)	CT scan	201 (140/61)	54%/43%	1316 (Pyradiomics)	Various univar test/Mutual information	Random forest	AUC = 0.790	AUC = 0.800
EGFR	-	MA J,W et al., Heliyon, 2024 (PMID: 38304841)	DESCT/CT scan	175 (122/53)	63%	1316 + 1209 transformed (Pyradiomics)	Corr filter/LASSO/MRMR	SVM	AUC = 0.780; ACC = 0.774; Se = 0.794; Sp	AUC = 0.830; ACC = 0.792; Se = 0.824; Sp

									= 0.737	= 0.737
EGFR	I–IV	Mu W et al., Nat. Commu., 2020 (PMID: 33067442)	18F-FDG PET/CT	618 (429/187) + (65)	47%/40%/55%	(CNN)	Deep learning	SResCNN	AUC = 0.810; ACC = 0.785	AUC = 0.840; ACC = 0.800
EGFR	I–IV	Ninomiya K et al., PLoS One, 2021 (PMID: 33428651)	CT scan	194 (99/95)	41%	54 (CNN Betti Number maps)	Univar test/Robustness index	SVM	AUC = 0.770; ACC = 0.710; Se = 0.720; Sp = 0.700	-
EGFR	-	Shang Y et al., La radiologia medica, 2023 (PMID: 37749461)	CT scan	779 (384/128/128)	54%/46%/25%	1454 (Pyradiomics)	Univar test/LASSO/MRMR	LGBM	AUC = 0.701; ACC = 0.621; Se = 0.660; Sp = 0.589	-
EGFR	I–IV	Shao X et al., BMC Med. Imaging, 2024 (PMID: 38438844)	18F-FDG PET/CT	516 (404/112)	61%	(CNN)	Deep learning	Models Genesis	AUC = 0.722; ACC = 0.661; Se = 0.676; Sp = 0.634	AUC = 0.730; ACC = 0.670; Se = 0.676; Sp = 0.659
EGFR	I–IV	Tu W et al., Lung Cancer, 2019 (PMID: 31097090)	CT scan	404 (243/161)	46%/46%	234 (MATLAB)	Corr filter/Univar test/Consensus clustering/Backward and step-wise selection	Logistic Regression	AUC = 0.775/ ACC = 0.677; Se = 0.878; Sp = 0.506	AUC = 0.818
EGFR	I–IV	Wang C et al., Front. Immunol., 2022 (PMID: 35250988)	CT scan	3629 (2903/726)	60%	1247 (RFs + CNN)	Deep learning/LASSO	Deep learning	AUC = 0.842; ACC = 0.763; Se = 0.797; Sp = 0.769	AUC = 0.895; ACC = 0.819; Se = 0.791; Sp = 0.850
EGFR	I–IV	Wang Y et al., Am. J. Nucl. Med. Mol. Imaging, 2024 (PMID: 38737644)	18F-FDG PET/CT	269 (189/80)	50%	(Pyradiomics)	Corr filter/Univar test	SVM	AUC = 0.768; ACC = 0.738; Se = 0.825; Sp = 0.650	AUC = 0.807; ACC = 0.775; Se = 0.775; Sp = 0.775
EGFR	I–IV	Weng Q et al., Front. Oncol., 2021 (PMID: 34422624)	CT scan	301 (210/91)	51%	396 (AK software)	LASSO/MRMR	Linear combination	AUC = 0.670; ACC = 0.720; Se = 0.670; Sp = 0.790	AUC = 0.750; ACC = 0.660; Se = 0.760; Sp = 0.570
EGFR	I	Wu J et al., Scientific Reports, 2024 (PMID: 38982267)	non-CE CT	438 (268/115) + (55)	64%/70%/71%	1834 (RFs)	Test-retest/Univar test/Corr filter/MRMR	SVM	AUC = 0.790	AUC = 0.809
EGFR	-	Xiao Z et al., Quant. Imaging Med. Surg., 2023 (PMID: 36915325)	18F-FDG PET/CT	150 (121/29)	43%/17%	(Deep learning)	Deep learning	EfficientNet-V2	AUC = 0.836; ACC = 81.92; F1 score =	-

								0.819	
EGFR	-	Xu N et al., J. Imaging Inform. Med., 2024 (PMID: 38361006)	CT scan	485 (339/146)	49%/49%	(Pyradiomics + deep learning)	Stepwise selection	EfficientNetV2-L-based + Radiomic (hybrid)	AUC = 0.941; ACC = 0.884	-
EGFR	I–IV	Yang B et al., Transl. Lung Cancer Res., 2020 (PMID: 32676320)	18F-FDG PET/CT	174 (139/35)	63%	1672 (Radiomics, Frontier, Siemens)	Random Forest importance	Random Forest	AUC = 0.710; Se = 0.810; Sp = 0.570	-
EGFR	I–IV	Yang L et al., Front. Oncol., 2022 (PMID: 35800046)	18F-FDG PET/CT	313 (218/95)	42%/42%	2074 (AK software)	ICC/Univar test/LASSO	SVM	AUC = 0.926	-
EGFR	-	Zhang B et al.,Front. Oncol., 2021 (PMID: 33643902)	CT scan	914 (709/205)	50%	1037 (Pyradiomics)	Deep learning	Squeeze-and-Excitation (SE) CNN model	AUC = 0.841; ACC = 0.775; Recall = 0.607; Prec = 0.796; F-score = 0.689	-
EGFR	-	Zhang G et al., Am. J. Cancer Res., 2021 (PMID: 33575086)	CT scan	420 (294/126)	50%/51%	1468 (Pyradiomics)	Univar test/LASSO/ICC	SVM	AUC = 0.796; ACC = 0.746; Se = 0.742; Sp = 0.750	AUC = 0.835; ACC = 0.802; Se = 0.773; Sp = 0.833
EGFR	I–IV	Zhang G et al., Quant. Imaging Med. Surg., 2024 (PMID: 39144003)	CT scan	660 (528/132)	49%	(Pyradiomics + CNN)	Univar test/Corr filter/ LASSO		AUC = 0.947; ACC = 0.938; Se = 0.938; Sp = 0.955	-
EGFR	I–IV	Zhang J et al., Eur. J. Nucl. Med. Mol. Imaging, 2020 (PMID: 31728587)	18F-FDG PET/CT	248 (175/73)	31%	92 (LifeX)	Univar test/LASSO	Logistic Regression	AUC = 0.85	AUC = 0.870
EGFR	I–IV	Zhao W et al., Cancer Med., 2019 (PMID: 31074592)	CT scan	579 (464/115) + (37)	53%	(RFs + CNN)	ICC/AutoML	3D DenseNets	AUC = 0.758; 0.750	-
EGFR	I-IV	Zhao W et al., Front. Oncol., 2020 (PMID: 31993370)	CT scan	637 (322/315)	53%/54%	475 (Matlab 2016b)	LASSO	Linear combination	AUC = 0.694	AUC = 0.734
EGFR	-	Zhao W et al., IEEE Trans. Neural Netw. Learn. Syst., 2024 (PMID: 35862326)	CT scan	640 (513/129) + (50)	54%	NA (Deep learning)	Deep learning	GMILT	AUC = 0.756; ACC = 0.760; Se = 0.750; Sp = 0.773	-

EGFR	I–III	Zhu H et al., Asia Pac. J. Clin. Oncol., 2022 (PMID: 35098682)	CT scan	1092 (875/217)	62%/62%	1078 (Pyradiomics)	Corr filter/LASSO	Logistic Regression	AUC = 0.646	AUC = 0.723
EGFR	I–IV	Zuo Y et al., Front. Oncol., 2023 (PMID: 37223682)	18F-FDG PET/CT	767 (410/177/88/92)	42%/44%/48%/28%	2380 PET and 2380 CT (Pyradiomics)	VAR/Corr filter/Mututal information	RFE + LGBM	AUC = 0.610; ACC = 0.550; (test cohort 2) AUC = 0.710; ACC = 0.700	-
EGFR	I–IV	Zuo Y et al., J. Cancer Res. Clin. Oncol., 2024 (PMID: 39436414)	18F-FDG PET/CT	566 (478/88)	68%/52%	NA (Pyradiomics)	ICC/Corr filter/Random forest	LGBM	AUC = 0.810; ACC = 0.570	-
EGFR/ALK	I–IV	Choe J et al., Eur. J. Radiol., 2021 (PMID: 33862316)	CT scan	503 (349/154)	EGFR: 38%/50%; ALK: 14%/17%	163 (Pyradiomics)	Random Forest/Corr filter	Logistic Regression	EGFR: AUC = 0.620; ALK: AUC = 0.680	-
EGFR/ALK/KRAS	-	Zhang X et al., Biomark. Res., 2024 (PMID: 38273398)	CT scan	508 (356/152)	39%	NA (RFs + CNN)	Deep learning/Rad-score	Random Forest + LDA	EGFR(RF): AUC = 0.884; ACC = 0.819; KRAS(LDA) AUC = 0.896; ACC = 0.803; ALK(RF) AUC = 0.884; ACC = 0.834	-
EGFR/KRAS	-	Dong Y et al., Quant. Imaging Med. Surg., 2021 (PMID: 34079707)	CT scan	525 (363/162)	EGFR: 45%/20%; KRAS: 23%/24%	NA (Pyradiomics + CNN)	NA	Multi-channel and multi-task deep learning (MMDL)	EGFR: AUC = 0.813; KRAS: AUC = 0.742	-
EGFR/KRAS	I–IV	Shiri I et al., Mol. Imaging Biol., 2020 (PMID: 32185618)	18F-FDG PET/CT	150 (82/68)	EGFR: 24%; KRAS: 24%	NA (Pyradiomics)	Various pre-selection tested/Univar test/Corr filter	Stochastic gradient descent (SGD)	EGFR: AUC = 0.820; KRAS: AUC = 0.830	-
EGFR/KRAS	I–IV	Velazquez E,R et al., Cancer Research, 2017 (PMID: 28566328)	CT scan	763 (353/352)	24%	NA (in-house developed Radiomics plug-in)	ICC/PCA/MRMR	Random forest	EGFR: AUC = 0.690; KRAS: AUC = 0.630	EGFR AUC = 0.750; KRAS AUC = 0.750
KRAS	-	Wang J et al., Front. Pharmacol., 2022 (PMID: 35431943)	18F-FDG PET/CT	258 (180/78)	50%/50%	NA (Pyradiomics)	Corr filter/Univar test/LASSO	Logistic Regression	AUC = 0.834; ACC = 0.782; Se = 0.923; Sp = 0.641	RFs + EGFR status: AUC = 0.921; ACC = 0.910; Se = 0.949; Sp =

										0.872

NOTE—Abbreviations: ACC: accuracy; AUC: area under the curve; AutoML: automated machine learning; BFE: backward feature elimination; CNN: convolutional neural network; Corr filter: correlation filter; DBSCAN: density-based spatial clustering of applications with noise; DESCT: dual-energy spectral computed tomography; GMILT: gated multiple instance learning transformer; ICC: intraclass correlation coefficient; LASSO: least absolute shrinkage and selection operator; LDA: linear discriminant analysis; LGBM: Light Gradient Boosting Machine; MRMR: minimum redundancy maximum relevance; PCA: principal component analysis; PET: positron emission tomography; Preci: precision; RF: radiomic feature; RFE: recursive feature elimination; Se: sensitivity; Sp: specificity; SVM: support vector machine; Univar test: significant univariate testing; VAR: variance filter; VIF: variance inflation factor; XGBoost: extreme gradient boosting
